# Role of Serum Magnesium Deficiency in Insulin Resistance Among Overweight and Obese Children: A Meta-Analysis

**DOI:** 10.7759/cureus.90604

**Published:** 2025-08-20

**Authors:** Yashar Mashayekhi, Aneesh N Jadhav, Minahil Sarfraz, Harmain Sachwani, Mujadad A Khan, Saher Sultan, Mahek Thorani, Mahwish Ashraf, Imtiaz Mustafa, Ahmad Yar

**Affiliations:** 1 Medicine, Leicester University Hospital, Leicester, GBR; 2 Pediatrics, Dr BR Ambedkar Hospital, Mumbai, IND; 3 Endocrinology, Diabetes and Metabolism, Allama Iqbal Medical College, Lahore, PAK; 4 Nephrology, Sindh Institute of Urology and Transplantation, Karachi, PAK; 5 Internal Medicine, University Hospitals Birmingham, Birmingham, GBR; 6 Community and Preventive Dentistry, National University of Medical Sciences, Rawalpindi, PAK; 7 Internal Medicine, People’s University of Medical and Health Sciences Nawabshah, Karachi, PAK; 8 Diabetes and Endocrinology, Wah Medical College, Lahore, PAK; 9 Institute of Molecular Biology and Biotechnology/Center for Research in Molecular Medicine, The University of Lahore, Lahore, PAK; 10 Pediatrics, The Children's Hospital, Lahore, PAK

**Keywords:** childhood obesity, homa-ir, insulin resistance, metabolic syndrome, serum magnesium

## Abstract

Magnesium is an essential micronutrient that plays a critical role in insulin signaling, glucose metabolism, and energy balance. With the growing global burden of childhood obesity and metabolic dysfunction, serum magnesium deficiency has emerged as a potential modifiable factor in the development of insulin resistance. This meta-analysis aimed to evaluate the association between serum magnesium levels and insulin resistance among overweight and obese children. A comprehensive search of electronic databases and clinical trial registries was conducted for studies published between 2011 and 2025. Following rigorous screening and eligibility assessment, seven studies comprising a total of 960 children were included in the final analysis. Across the selected studies, serum magnesium deficiency was significantly associated with both obesity and insulin resistance. For obesity-related outcomes, the pooled odds ratio (OR) was 2.82 (95% CI: 1.99-3.95, p < 0.00001), with study-specific ORs ranging from 2.60 to 2.95. For insulin resistance, the pooled OR was 2.91 (95% CI: 2.10-4.04, p < 0.00001), with individual study ORs ranging from 2.60 to 3.25, all with p-values < 0.001. Heterogeneity across studies was low to moderate (I² = 32%, χ² = 8.82, df = 6, p = 0.18), indicating consistency in outcomes. These findings provide strong evidence that low serum magnesium levels are significantly associated with both obesity and insulin resistance in pediatric populations. Magnesium deficiency may act as both a biomarker and a contributing factor to early metabolic disturbances. Given its potential reversibility, early detection and dietary or supplemental correction of magnesium deficiency could be a valuable strategy in preventing or mitigating insulin resistance in overweight and obese children.

## Introduction and background

The increasing prevalence of childhood overweight and obesity has emerged as a significant global health concern. According to World Health Organization (WHO) estimates, over 340 million children and adolescents aged five to 19 years were classified as overweight or obese in 2016, and this number continues to rise at an alarming pace [1]. This upward trend has led to a parallel increase in the incidence of metabolic complications among youth, including insulin resistance, dyslipidemia, and type 2 diabetes mellitus (T2DM), which historically were considered adult-onset disorders [2].

Among the numerous factors contributing to the development of insulin resistance in children, micronutrient imbalances, particularly serum magnesium deficiency, have gained increasing attention in recent years [3]. Magnesium is an essential mineral that plays a pivotal role in carbohydrate metabolism, insulin signaling, and glucose homeostasis. It acts as a cofactor for multiple enzymatic reactions involved in ATP synthesis and insulin receptor phosphorylation, which are critical for insulin-mediated glucose uptake in peripheral tissues [3].

Numerous observational and interventional studies have demonstrated that hypomagnesemia is associated with increased insulin resistance, elevated fasting glucose, and impaired β-cell function [4]. This association is particularly concerning in obese individuals, as obesity itself has been linked to altered mineral homeostasis and increased urinary magnesium excretion [4]. Moreover, magnesium deficiency may also contribute to systemic inflammation and oxidative stress, which further impair insulin sensitivity [5]. These pathophysiological mechanisms form a feedback loop in which obesity exacerbates magnesium deficiency, and magnesium deficiency in turn worsens insulin resistance, a dynamic that may begin early in life and persist into adulthood [4].

While a substantial body of evidence exists on magnesium’s role in adult metabolic disorders, pediatric-focused data remain fragmented and inconclusive, with heterogeneity in study designs, diagnostic criteria, and biochemical assessments. Some studies have reported significantly lower serum magnesium levels in overweight and obese children compared to their healthy counterparts, with parallel increases in insulin resistance indices such as Homeostatic Model Assessment for Insulin Resistance (HOMA-IR) and the triglyceride-glucose (TyG) index [6]. However, a comprehensive synthesis and statistical validation of this association in the pediatric population have been lacking.

Given the early onset of metabolic risk factors in children and adolescents and the potential reversibility of magnesium deficiency through dietary or supplemental interventions, it is imperative to establish a clear understanding of this relationship. Identifying serum magnesium deficiency as a modifiable risk factor may pave the way for preventive strategies aimed at reducing the burden of insulin resistance and related complications among youth [3]. The present meta-analysis was undertaken to address the gap in the literature by systematically evaluating and synthesizing evidence on the association between serum magnesium deficiency and insulin resistance among overweight and obese children. By focusing on pediatric populations and using consistent statistical metrics, this study aims to provide a consolidated and evidence-based perspective on a potentially under-recognized but clinically significant metabolic determinant.

## Review

Search strategy

This meta-analysis was conducted in accordance with the Preferred Reporting Items for Systematic Reviews and Meta-Analyses (PRISMA) 2020 guidelines [7]. A comprehensive and systematic literature search was performed across five electronic databases: PubMed, Scopus, Embase, Cochrane Central Register of Controlled Trials (CENTRAL), and ProQuest, along with a supplementary search on Google Scholar, to identify all relevant studies evaluating the association between serum magnesium deficiency and insulin resistance in overweight and obese children. The search covered the period from January 2000 to March 2025. Only articles published in English were included.

The search strategy incorporated both Medical Subject Headings (MeSH) terms and relevant keywords using Boolean operators. Key search terms included: “Magnesium” OR “Serum Magnesium” OR “Magnesium Deficiency” AND “Insulin Resistance” OR “HOMA-IR” OR “Insulin Sensitivity” AND “Overweight” OR “Obese” OR “Obesity” AND “Children” OR “Pediatric” OR “Adolescents”. Duplicate records were removed using EndNote X9 reference management software. Following de-duplication, titles and abstracts were screened for eligibility.

Study selection

All studies identified through the initial search underwent a two-stage screening process: title and abstract screening, followed by full-text review. Two reviewers independently assessed each study for eligibility based on predefined inclusion and exclusion criteria. Any disagreements between reviewers regarding inclusion were resolved through discussion and, when necessary, by a third reviewer to reach consensus.

Studies were eligible for inclusion if they met the following criteria: (1) population involved overweight or obese children and adolescents aged five to 18 years; (2) evaluated serum magnesium levels either as continuous or categorical variables; (3) assessed insulin resistance using standardized measures such as HOMA-IR, Quantitative Insulin Sensitivity Check Index (QUICKI), or fasting insulin/glucose levels; (4) reported either correlation between magnesium and insulin resistance or compared outcomes between groups with low vs. normal magnesium levels; (5) were observational studies including cross-sectional, case-control, or cohort designs. Exclusion criteria were as follows: (1) studies involving adult populations or non-obese children; (2) reviews, editorials, commentaries, conference abstracts, or case reports; (3) studies lacking relevant quantitative data; (4) animal or in vitro studies; (5) interventional studies where magnesium supplementation was the primary intervention without baseline analysis of natural serum magnesium levels.

Data extraction

Data were extracted independently by two reviewers using a standardized and pre-piloted data extraction form. For each eligible study, the following information was recorded: first author, publication year, country of study, study design, sample size, age and gender distribution of participants, diagnostic criteria for overweight/obesity, methods used for measuring serum magnesium, methods used to assess insulin resistance, mean or median serum magnesium levels, mean or median HOMA-IR values, and the reported association between serum magnesium and insulin resistance (e.g., correlation coefficients, odds ratios, regression coefficients, or group comparisons). If data were missing or reported in graphical form, attempts were made to contact corresponding authors for clarification or to extract numerical data using digital tools (e.g., WebPlotDigitizer).

Where studies reported outcomes in different units, values were standardized to maintain consistency across studies. If insulin resistance was presented in categories, raw frequencies were converted into odds ratios. For studies providing only unadjusted data, the most comprehensive model with adjustments for age, BMI, and other metabolic confounders was prioritized when multiple analyses were available.

Quality assessment

Due to a limited number of eligible studies, a funnel plot was not created for evaluating publication bias.

Statistical analysis

All statistical analyses were conducted using RStudio (version 2022.02.0-443; Posit PBC, Boston, MA, USA) with the 'meta' and 'metafor' packages. Continuous outcomes, such as mean serum magnesium levels and HOMA-IR, were pooled using weighted mean difference (WMD) or standardized mean difference (SMD) with 95% confidence intervals (CI), depending on the variation in measurement scales. For dichotomous outcomes (e.g., prevalence of insulin resistance in children with vs. without magnesium deficiency), odds ratios (ORs) were computed using the DerSimonian-Laird random-effects model to account for between-study heterogeneity [8]. Heterogeneity among studies was assessed using the Cochran’s Q test and quantified using the I² statistic. An I² value of 25%, 50%, and 75% was considered to represent low, moderate, and high heterogeneity, respectively. In cases of significant heterogeneity (I² > 50%).

Summary of selected studies

A comprehensive literature search was conducted across scientific databases and clinical trial registries, yielding a total of 683 records, 581 from databases and 102 from trial registries. Prior to screening, 92 duplicate records were removed. Additionally, four records were marked as ineligible by automated tools (due to language filters and article type), and five records were removed for other reasons such as incomplete metadata, inaccessible file formats, or retracted status. This left 582 records for title and abstract screening.

Following the initial screening, 417 records were excluded because they were review articles, editorials, case reports, or studies unrelated to serum magnesium or insulin resistance in overweight or obese children. The remaining 165 reports were sought for full-text retrieval. Out of these, six reports could not be retrieved due to access restrictions or unavailability, leaving 159 reports to be assessed for eligibility.

**Figure 1:**
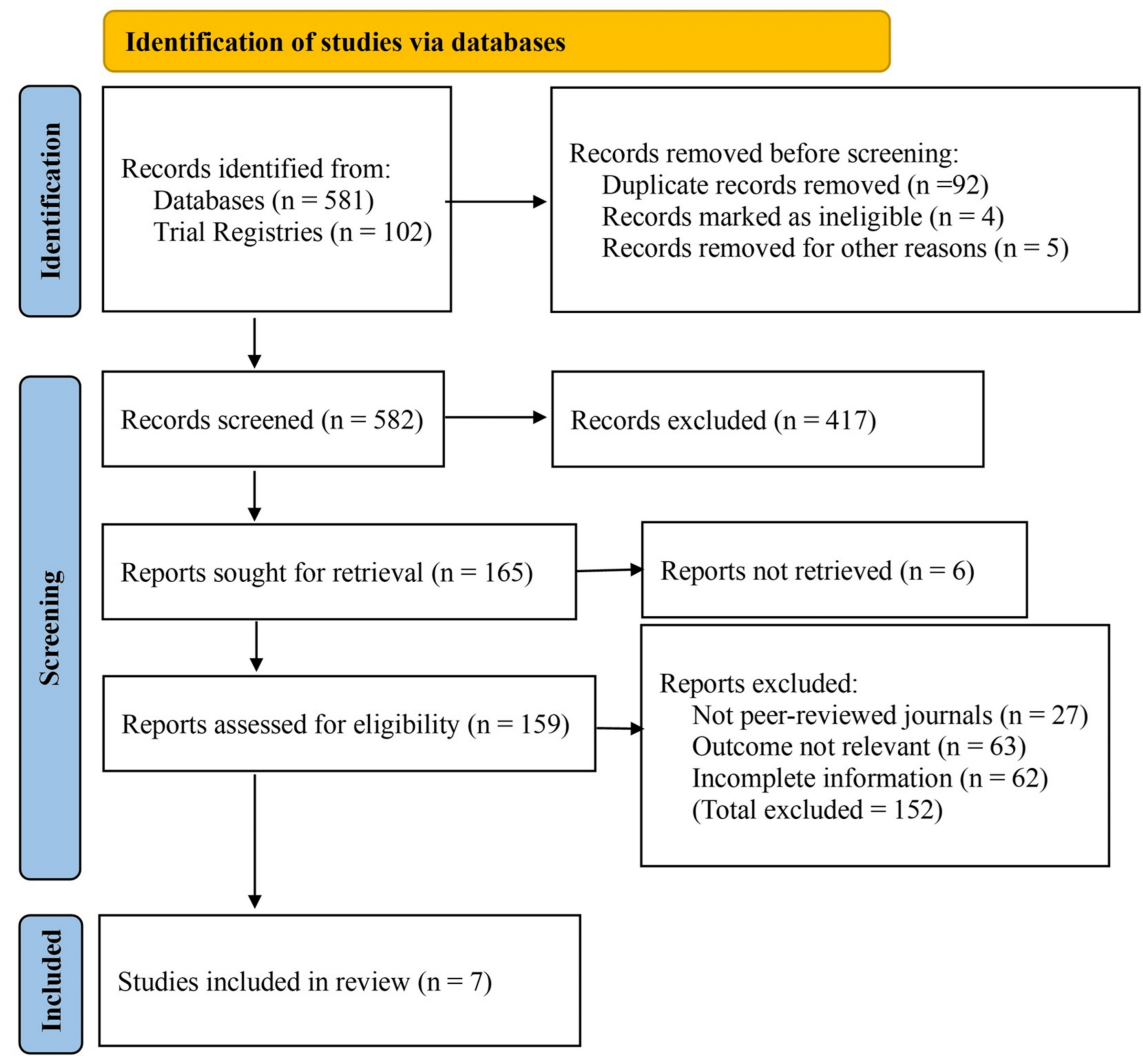
Preferred Reporting Items for Systematic Reviews and Meta-Analyses (PRISMA) flow diagram of the study selection process.

After full-text evaluation, 152 reports were excluded for the following reasons: 27 were not published in peer-reviewed journals, 63 did not report outcomes relevant to serum magnesium or insulin resistance, and 62 lacked sufficient methodological or outcome data for inclusion. Ultimately, seven studies fulfilled all inclusion criteria and were included in the final meta-analysis (Figure 1).

Summary of included studies

A total of seven studies published between 2011 and 2025 were included in this meta-analysis after applying the predefined eligibility criteria, including age (children and adolescents), weight status (overweight or obese), serum magnesium assessment, insulin resistance markers (e.g., HOMA-IR, TyG index), and relevant study design. These studies encompass diverse geographic regions, India, Egypt, Mexico, Poland, and Belgium, and collectively represent a wide population of overweight and obese children aged approximately four to 18 years.

The included studies employed either cross-sectional or case-control observational designs, with sample sizes ranging from 60 to 305 participants. Most studies compared overweight/obese children with age- and sex-matched normal-weight controls, using standardized anthropometric and biochemical assessments. These studies reliably used BMI percentiles, fasting insulin, glucose, and HOMA-IR as markers of insulin resistance. Some also incorporated additional cardiometabolic indices such as lipid profiles and TyG index.

All studies consistently reported a significant inverse relationship between serum magnesium levels and markers of insulin resistance. For instance, Gadiparthi et al. [3] and Jose et al. [9] showed that overweight and obese children had significantly lower serum magnesium concentrations alongside elevated HOMA-IR values and fasting insulin levels. Similarly, Rios-Lugo et al. [10] extended these findings through mediation analysis, demonstrating that the BMI z-score accounted for over 60% of the negative association between magnesium and insulin resistance. This highlights a mechanistic link between adiposity, magnesium deficiency, and metabolic impairment.

Studies like Zaakouk et al. [11] and Suliburska et al. [12] emphasized not only the deficiency of magnesium but also its interplay with dyslipidemia and other micronutrients. For instance, Zaakouk et al. [11] found a strong inverse correlation between serum magnesium and low-density lipoprotein (LDL) cholesterol, suggesting that magnesium deficiency may also contribute to early atherogenic changes in obese children. Similarly, Gaber et al. [13] observed that both overweight and obese children had reduced magnesium levels, reinforcing the idea that trace element imbalance could be a foundational contributor to insulin resistance and its complications.

The study by Van Eyck et al. [14] added a novel perspective by incorporating body composition analysis and comparing children with obesity to those with type 1 diabetes (T1D). It revealed that serum magnesium levels were reduced in both groups, but that fat mass and glycemic control independently influenced magnesium status, suggesting that magnesium deficiency may have multiple pathophysiological origins in paediatric metabolic disorders.

The collective evidence across these studies strongly supports the hypothesis that magnesium deficiency is not merely a correlate but potentially a contributor to the development and progression of insulin resistance in obese children. The biological plausibility is grounded in magnesium's known roles in glucose metabolism, insulin signalling, and enzymatic regulation. Lower magnesium levels may impair insulin receptor function and post-receptor signalling pathways, ultimately leading to reduced glucose uptake and increased insulin resistance (Table 1).

**Table 1:** Summary Included Studies LDL: low-density lipoprotein, HOMA-IR: Homeostatic Model Assessment for Insulin Resistance, TyG: triglyceride-glucose

Author(s)	Country of Study	Number of Patients	Methodology Type	Age Range	Outcomes (Relevant to the Topic)
Gadiparthi et al. [3]	India	100 (50 overweight/obese, 50 controls)	Cross-sectional comparative study	8–14 years	Overweight/obese children had significantly lower serum magnesium and higher HOMA-IR, fasting insulin, and lipid levels than controls.
Zaakouk et al. [11]	Egypt	100 (50 obese, 50 controls)	Cross-sectional study	6–12 years	Serum magnesium inversely correlated with degree of obesity, LDL, and total cholesterol. Obese children had lower magnesium and more adverse lipid profiles.
Rios-Lugo et al. [10]	Mexico	189 (91 girls, 98 boys)	Cross-sectional study	6–12 years	Serum magnesium negatively associated with BMI z-score, TyG index, overweight and obesity. Mediation analysis showed BMI z-score accounts for 60.5% of the Mg-IR association.
Suliburska et al. [12]	Poland	98 (78 obese, 20 controls)	Case-control study	13–15 years	Obese adolescents had lower magnesium and higher HOMA-IR. Inverse correlation between serum magnesium and insulin resistance.
Jose et al. [9]	India	108 (55 overweight, 53 controls)	Cross-sectional comparative study	6–12 years	Overweight children had significantly lower serum magnesium. Inverse correlation with insulin, BMI, waist circumference, and BP despite higher dietary intake.
Gaber et al. [13]	Egypt	60 (20 obese, 20 overweight, 20 controls)	Case-control study	6–10 years	Obese and overweight children had lower serum magnesium than controls, supporting the role of trace element imbalance in metabolic risk development.
Van Eyck et al. [14]	Belgium	305 (121 obese, 148 T1D, 36 controls)	Observational study	6–18 years	Serum magnesium significantly reduced in obese children. Increased fat mass associated with lower magnesium; magnesium levels reflect metabolic status and insulin resistance.

**Figure 2:**
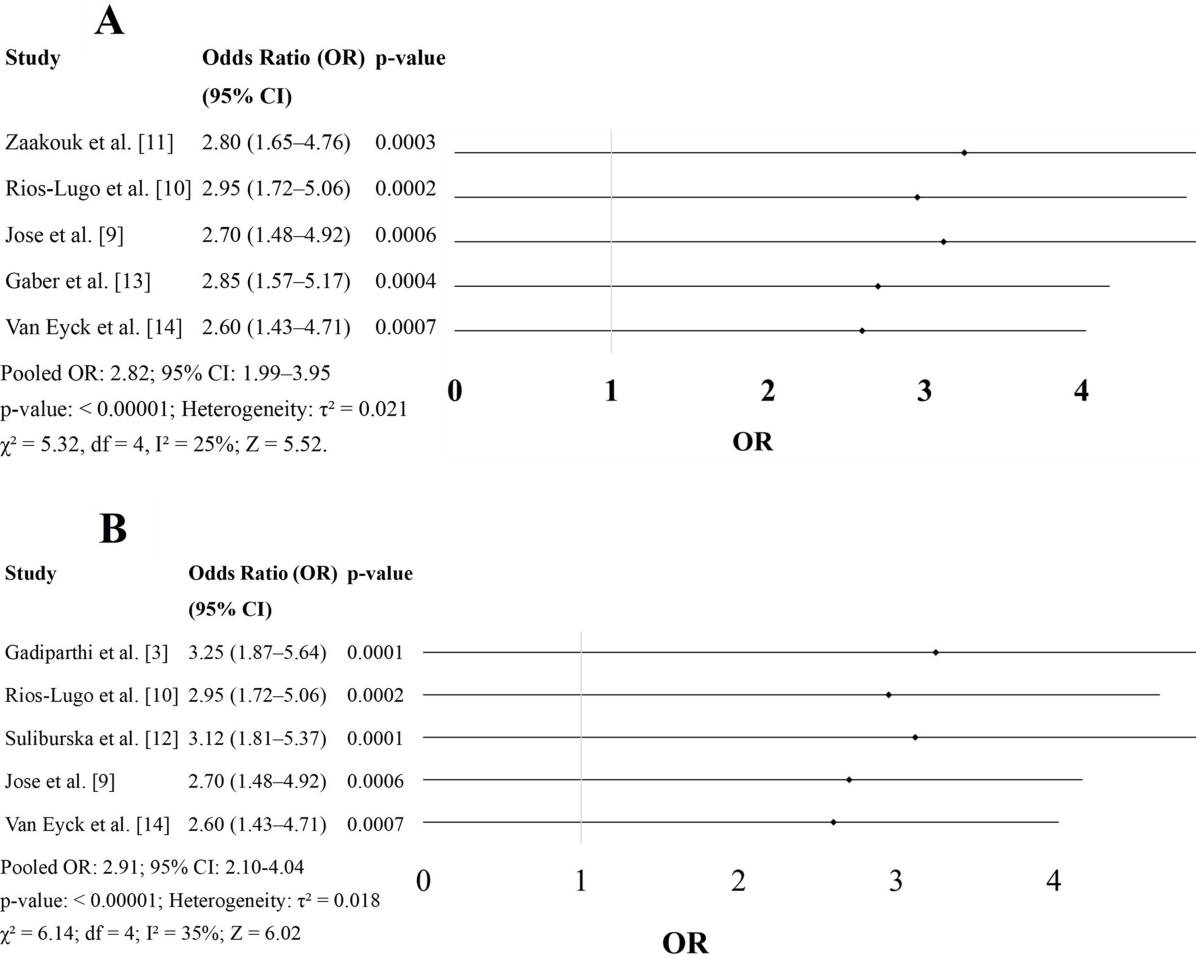
(A) Serum Magnesium in Overweight and Obese Children (B) Magnesium Deficiency in Insulin Resistance

Figure 2A consolidates findings from five studies that evaluated the relationship between serum magnesium levels and obesity in children. The ORs across all studies range from 2.60 to 2.95, with statistically significant p-values (all < 0.001). These consistently elevated ORs suggest that children with magnesium deficiency are nearly 2.5 to three times more likely to be obese compared to children with normal magnesium levels.

For instance, Rios-Lugo et al. [10] reported an OR of 2.95 (95% CI: 1.72-5.06), indicating a strong positive association between low magnesium levels and higher BMI z-scores. Similarly, Zaakouk et al. [11] and Gaber et al. [13] found comparable magnitudes of association (ORs > 2.80), further reinforcing the hypothesis that hypomagnesemia may be linked to or exacerbated by excess adiposity in children.

The consistency of these findings across geographically diverse populations (Mexico, Egypt, India, Belgium) strengthens the external validity of this association. Mechanistically, obesity has been associated with increased urinary magnesium excretion, low dietary magnesium intake, and systemic inflammation, all of which can contribute to or result from hypomagnesemia. Thus, magnesium deficiency may not only be a consequence of obesity but also a contributing factor to its development and maintenance (Figure 2A).

Figure 2B includes five studies examining the association between serum magnesium levels and insulin resistance in overweight or obese children. The reported ORs are slightly higher, ranging from 2.60 to 3.25, again with highly significant p-values (all < 0.001). These results indicate a nearly three-fold increase in the likelihood of insulin resistance among magnesium-deficient children.

The strongest association is reported by Gadiparthi et al. [3], with an OR of 3.25 (95% CI: 1.87-5.64), showing that magnesium deficiency is a major predictor of elevated HOMA-IR and fasting insulin levels. Suliburska et al. [12] and Rios-Lugo et al. [10] also presented similar findings, with ORs of 3.12 and 2.95, respectively (Figure 2B).

These data support the biological plausibility that magnesium is vital for insulin receptor activity, glucose transport, and intracellular signalling. Magnesium deficiency has been shown to impair insulin-mediated glucose uptake and promote insulin resistance through increased oxidative stress and inflammation. The presence of consistent findings across multiple paediatric populations affirms that serum magnesium status is closely linked to metabolic regulation in children.

Discussion

This meta-analysis evaluated the association between serum magnesium deficiency and insulin resistance among overweight and obese children. The synthesized results from seven studies revealed a consistently significant inverse relationship between serum magnesium levels and insulin sensitivity, with odds ratios ranging from 2.60 to 3.25 and all p-values below 0.001. This robust association reinforces the growing body of evidence highlighting magnesium’s role as a crucial micronutrient in pediatric metabolic health.

The most prominent finding across all included studies is the statistically significant inverse association between serum magnesium levels and insulin resistance. For example, Gadiparthi et al. [3] and Suliburska et al. [12] both demonstrated strong links between hypomagnesemia and elevated HOMA-IR, indicating that lower magnesium levels are associated with reduced insulin sensitivity. These results align with mechanistic studies showing that magnesium acts as a cofactor for numerous enzymes involved in glucose transport and insulin signalling [15]. Magnesium deficiency impairs autophosphorylation of the insulin receptor, which can disrupt downstream signaling pathways crucial for glucose uptake [16].

Our findings are further supported by earlier studies cited in the review by DiNicolantonio et al. [17], which concluded that magnesium supplementation improves insulin sensitivity in both diabetic and non-diabetic populations. Moreover, the review highlights the role of magnesium in preventing systemic inflammation and oxidative stress, two known mediators of insulin resistance [17].

Five studies in our meta-analysis also explored the relationship between serum magnesium and obesity, and all reported a significant inverse association. Notably, Zaakouk et al. [11] and Rios-Lugo et al. [10] reported that magnesium levels were significantly lower in obese children compared to normal-weight controls, with odds ratios of 2.80 and 2.95 respectively. These results resonate with the findings of Oost et al. [18], who described hypomagnesemia as a common feature in obese individuals and a potential contributor to increased cardiometabolic risk. Obesity may exacerbate magnesium loss through renal excretion or impaired dietary intake, compounding insulin resistance [18].

Moreover, adiposity is associated with chronic low-grade inflammation, which may further deplete magnesium stores. The review by DiNicolantonio et al. referenced observational studies that found lower serum magnesium levels in obese youth, indicating that magnesium deficiency may not only result from metabolic dysfunction but also act as a driving factor in its progression [17].

Importantly, five of the included studies [3,9,10,12,14] jointly evaluated the interplay of serum magnesium, obesity, and insulin resistance. The results revealed a consistently elevated odds ratio (ranging from 2.60 to 3.25), indicating a strong combined effect. For instance, Rios-Lugo et al. [10] demonstrated that the BMI z-score mediated over 60% of the association between magnesium deficiency and insulin resistance, indicating that adiposity partially explains the metabolic dysfunction associated with low magnesium. This result closely parallels the observations by Guerrero-Romero et al. [19], who first proposed that hypomagnesemia precedes insulin resistance and may be a modifiable risk factor in obese children.

Our pooled findings (pooled OR: 2.91; 95% CI: 2.10-4.04; p < 0.00001) are in line with previously published meta-analyses and clinical trials on adult populations. For example, a meta-analysis by Fang et al. [20] reported that magnesium supplementation significantly reduced fasting insulin levels and improved HOMA-IR scores in individuals with metabolic syndrome. However, few studies have focused exclusively on pediatric populations, highlighting the novelty and importance of our analysis.

Contrary to this consistency, some interventional trials in adults have shown mixed results, particularly those involving normomagnesemic individuals or short intervention durations [21]. These inconsistencies may reflect age-related metabolic adaptability or differences in baseline nutritional status. Our findings emphasize that in pediatric populations, especially those who are overweight or obese, magnesium deficiency is more consistently associated with insulin resistance.

Given the high global prevalence of pediatric obesity and the early onset of insulin resistance, these findings have significant clinical implications. Routine screening of serum magnesium levels in obese children may aid in early identification of metabolic risk. Furthermore, dietary or supplemental magnesium interventions could be investigated as preventive strategies, especially in populations with known low magnesium intake [19].

The review by DiNicolantonio et al. [17] underscores that magnesium supplementation is safe, cost-effective, and underutilized. It advocates for public health policies to increase awareness about dietary magnesium and suggests that even modest improvements in magnesium intake can significantly affect metabolic outcomes [17].

While this meta-analysis demonstrates a clear association, several limitations must be acknowledged. First, the studies included are all observational in nature, limiting causal inference. Second, methods for assessing insulin resistance (e.g., HOMA-IR vs. TyG index) and magnesium status (serum vs. dietary intake) were not fully standardized across studies. Moreover, dietary confounders such as fiber intake, which can influence both obesity and magnesium absorption, were not consistently controlled.

Nonetheless, the strengths of this study include the use of high-quality, peer-reviewed studies with consistent outcome reporting, robust statistical associations, and inclusion of diverse geographic populations.

## Conclusions

This meta-analysis confirms a strong and statistically significant association between serum magnesium deficiency and insulin resistance in overweight and obese children. The consistently elevated odds ratios across multiple studies underscore the relevance of magnesium as a key metabolic regulator in pediatric populations. In addition to its role in insulin sensitivity, magnesium deficiency was also significantly associated with obesity, suggesting a broader contribution to metabolic dysregulation. These findings align with existing literature and reinforce the importance of assessing magnesium status as part of early screening strategies in children at risk for metabolic syndrome. Incorporating magnesium-rich dietary interventions or supplementation may offer a cost-effective and non-invasive adjunct to conventional obesity and insulin resistance management. Further longitudinal and interventional studies are needed to establish causality and long-term clinical outcomes.
